# Medical Selections from Herndon’s Report of the Amazon

**Published:** 1854-07

**Authors:** 


### *Medical Selections from Herndon's Report of the Amazon.*

The following contains some curious incidents, which will be read with interest, particularly, that relating to the use and effects of the "*parica.*" The effect was truly astonishing as well as happy. The impression seems to be made upon the brain and then radiating from that centre throughout the entire system :—

"The precious and medicinal guarana plant, which the Brazilians of the central provinces of Goyaz and Matto Grosso purchase with its weight in gold, to use against the putrid fevers which rage at certain periods of the year, is owed to the Mahues Indians. They alone know how to prepare it, and entirely monopolize it.

The Tuchao of the Malocca, called Mandu-assu, received me with cordiality and offered me his cabin. Fatigued from the journey, and finding there some birds and rare plants, I remained several days.

Mandu-assu marvelled to see me carefully preserve the birds the hunter killed, and the leaves of plants, or wood, that possessed medicinal virtues. He never left me; accompanied me through the forests, and gave me many plants of whose properties I was ignorant.

Rendered still more communicative by the small presents I made him, he gave me not only all the particulars I wished upon the cultivation and preparation of the guarana, but also answered fully all my questions.

I left him for the Malocca of *Mosse*, whose chief was his relative. This chief was more distant and savage than Mandu-assu, and received me with suspicion. I was not discouraged, as I only went to induce him to exchange, for some articles, his *parica*, or complete apparatus for taking a kind of snuff which the great people of the country frequently use.

My cause, however, was not altogether lost; my hunter who had been in a cabin of the village, took me to see a young Indian who had been bitten the evening previous by a *surucucurano* serpent. I opened the wound, bled him, and again used the volatile salts. Whilst I operated, a young Indian woman, singularly beautiful, sister of the wounded man, supported the leg. She watched me with astonishment, and, whilst I was binding up the wound with cotton soaked in alkali, (salts,) she disappeared, and I saw her no more.

The Indian was relieved. The old Tuchao knew of it; and, to thank me for it, or rather, I believe, to test me, presented me with a calabash, in which he poured a whitish and disgusting drink, exhaling a strong odor of corruption. This detestable liquor was the *cachiri*, (masato,) a drink that would make hell vomit; but the Indians passionately love it. I knew by experience that by refusing to drink I would offend this proud Mahue, and that if I remained in this Malocca I should assuredly die from want, because even a calabash of water would be refused me. I shut my eyes and drank.

The cachiri is the substance of the manioc root, softened in hot water, and afterwards chewed by the old women of the Malocca.—They spit it into great earthern pans, when it is exposed to a brisk fire until it boils. It is then poured into pots and suffered to stand until a putrid fermentation takes place.

The Indian afterwards took his parica. He beat, in a mortar of sapucaia, a piece of hard paste, which is kept in a box made of shell; poured this pulverized powder upon a dish presented by another Indian, and with a long pencil of hairs of the *tamandua bandeira*, he spread it evenly without touching it with the fingers; then taking pipes joined together, made of the quills of the *gaviao real*, (royal eagle,) and placing it under his nose, he snuffed up with a strong inspiration all the powder contained in the plate. His eyes started from his head; his mouth contracted; his limbs trembled. It was fearful to see him; he was obliged to sit down, or he would have fallen; he was drunk, but this intoxication lasted but five minutes; he was then gayer.

Afterwards, by many entreaties, I obtained from him his precious parica, or rather one of them, for he possessed two.

At the Malocca of *Taguariti*, where I was the next day, the Tuchao, observing two young children returning from the woods laden with sarsaparilla, covered with perspiration, and overcome, as much by the burden they carried as the distance they had traveled, called them to him, beat some parica, and compelled them to snuff it.

I then understood that a Tuchao Mahue had a paternal authority in his Malocca, and treated all as his own children. He forced these children to take the parica, convinced that by it they avoided fevers and other diseases. And, in truth, I soon saw the children leave the cabin entirely refreshed, and run playing to the brook and throw themselves in.

Several vegetable substances compose parica: first, the ashes of a vine that I cannot class, not having been able to procure the flowers; second, seeds of the *acacia angico*, of the luguminous family; third, juice of the leaves of the *abuta*, (cocculus) of the menispermis family.

I never saw a Mahue Indian sick, nor even heard them complain of the slightest pain, notwithstanding that the forests they inhabit are the birthplaces of dangerous fevers, which rarely spare the Brazilian merchants who come to purchase sarsaparilla roots."

The following is a severe trial of manhood surely. We wot of certain "Benedicts" who have some latent inclination to enter into the "connubial state," but who, if they had to pass such an ordeal would surrender all hope and crush every uprising disposition or inclination :—

"An Indian is not a renowned Mahue, and cannot take a wife, until he has passed his arms at least ten times through long stalks of the palm-tree, filled intentionally with large, venomous ants. He whom I saw receive this terrible baptism was not sixteen years old. They conducted him to the chiefs, where the intruments awaited him; and, when muffled in these terrible mittens, he was obliged to sing and dance before every cabin of the Malocca, accompanied by music still more horrible. Soon the torments he endured became so great that he staggered. (The father and relatives dread, as the greatest dishonor that can befall the family, a cry or a weakness on the part of the young martyr. They encourage and support him, often by dancing at his side.) At length he came to the last cabin; he was pallid; his teeth chattered; his arms were swollen; he went to lay the gloves before the old chief, where he still had to endure the congratulations of all the Indians of the Malocca. Even the young girls mercilessly embraced him, and dragged him through all their circles; but the Indian, insensible to their caresses, sought only one thing—to escape. At length he succeeded, and, throwing himself into the stream, remained there until night.

The *Tocandeira* ants not only bite, but are also armed with a sting like the wasp; but the pain felt from it is more violent. I think it equal to that occasioned by the sting of the black scorpion.

In one of my excursions in the environs of the Malocca of Manduassu, I had occasion to take several of them. I enclosed them in a small tin box. I afterwards let one bite me, that I might judge in a slight degree what it costs the young Mahues to render themselves acceptable. I was bitten at 10 a. m. I felt an acute pain from it until evening, and had several hours' fever."

Upon one of the tributaries of the Amozon is the great diamond region. It is, however, very unhealthy. The question will arise why is it so? The diamond is pure carbon and in the regions in which it is found are there not carboniferous emanations:

"It would appear, however, that diamond-hunting is not as productive as it is believed; for they quote in the country, as very remarkable, the result obtained by a Spaniard, Don Simon by name, who, in four years (only working, it is true, during the dry season, but with two hundred slaves) had collected four hundred oitavas of diamonds, (about seven thousand carats.) He was obliged to abandon the work because he lost many slaves in consequence of the pestilential fevers which reign in the diamond region, and particularly upon the borders of the river Santa Anna. Before his departure, he filled up the place from whence he extracted the stones.

I do not doubt that this region may one day furnish, if it is submitted to a well-conducted exploration, an infinitely larger quantity. Unfortuuately, as we have already said, the search for these stones is accompanied with great danger; and I am convinced that these baubles of human vanity have already cost, to Brazil alone, the life of more than a hundred thousand human beings."

The following described institution serves one good purpose, viz: that of preventing the exposure of certain little specimens of "mortality" from being exposed upon the door steps of the mansions of the rich. which is often but the gathering of the "bread which had been thrown upon the waters," or "stray chicks coming home to the paternal roost."

"There are several hospitals and charitable institutions in the city, (Para) among which is a very singular one. This is a place for the reception of foundlings maintained by the city. A cylinder, with a reecptacle in it sufficiently large for the reception of a baby, turns upon an axis in a window; any one may come under cover of night, deposit a child in the cylinder, turn the mouth of the receptacle in, and walk away without being seen. Nurses are provided to take charge of the foundling.

Though I pumped all my acquaintances, I could get no statistics concerning this institution, or whether it was thought to be beneficial or not. I judge, however, that for this country it is. Public opinion here does not condemn, or at least treats very leniently, the sins of fornication and adultery. This institution, therefore, while it would tend to lessen the crime of infanticide, would not encourage the above-mentioned sins by concealment; for where there is no shame there is no necessity for concealment. In speaking thus, I do not at all allude to the higher classes of Bazil. (No, no, no blame ever attached to them.—Ed.)

The following curious circumstances, if true, would militate seriously against the theory of the unity of the races. The naturalist will find here a field of research and investigation. The discovery of that African race which burrows in the ground, of short stature, and living upon insects, but in perfect proportions, would go very far toward establishing the fact of a comtinued and almost unbroken chain of being, mingling and blending and rising upward in imperceptible degrees :—

"M. Castelnau collected some very curious stories concerning the Indians who dwell upon the banks of the Jurua. He says, (vol. 5. p. 105,) "I cannot pass over in silence a very curious passage of Padre Noronha, and which one is astonished to find in a work of a character in other respects. The Indians, *Cauamas* and *Uginas*, (says the padre,) live near the sources of the river. The first are of very short stature, scarcely exceeding five palms, (about three and a half feet;) and the last (of this there is no doubt) have tails, and are produced by a mixture of Indians and *Coata* monkeys. Whatever may be the cause of this fact, I am led to give it credit for three reasons: first, because there is no physical reason why men should not have tails; secondly, because many Indians, whom I have interrogated regarding this thing, have assured me of the fact, telling me that the tail was a palm and a half long; and, thirdly, because the Reverend Father Friar Jose de Santa Theresa Ribeiro, a Carmelite, and Curate of Castro de Avelaens, assured me that he saw the same thing in an Indian who came from Japura, and who sent me the following attestation :

'I, Jose de Santa Theresa Ribeiro, of the Order of our Lady of Mount Carmel, Ancient Observance, &c., certify and swear, in my quality of Priest, and on the Holy Evangelists, that when I was a missionary in the ancient village of Parauau, where was afterwards built the village of Noguera, I saw, in 1755, a man called Manuel da Silva, native of Pernumbuco, or Bahia, who came from the river Japura with some Indians, amongst whom was one—an Infidel brute —who the said Manuel declared to me had a tail; and as I was unwilling to believe such an extraordinary fact, he brought the Indian and caused him to strip, on pretence of removing some turtles from a 'pen,' near which I stood to assure myself of the truth. There I saw, without possibility of error, that the man had a tail, of the thickness of a finger, and half a palm long, and covered with a smooth and naked skin. The same Manuel assured me that the Indian had told him that every month he cut his tail, because he did not like to have it too long, and it grew very fast. I do not know to what nation this man belonged, nor if all his tribe had a similar tail; but I understood afterwards that there was a tailed nation upon the banks of the Jurua; and I sign this act and seal it in affirmation of the truth of all that it contains.

ESTABLISHMENT OF CASTRO DE AVELAENS, October 14, 1768.

FR. JOSE DE STA. THERESA RIBEIRO.'

M. Baena (Corog, Para) has thought proper to repeat these strange assertions. 'In this river,' says he, speaking of the Jurua, (p. 487,) 'there are Indians, called Canamas, whose height does not exceed five palms; and there are others, called Uginas, who have a tail of three or four palms, (four palms and an inch, Portuguese, make nearly an English yard,) according to the report of many persons. But I leave to every one to put what faith he pleases in these assertions.'

M. Castelnau says, after giving these relations, 'I will add but a word. Descending the Amazon, I saw one day, near Fonteboa, a black Coata, of enormous dimensions. He belonged to an Indian woman, to whom I offered a large price, for the country, for the curious beast; but she refused me with a burst of laughter. 'Your efforts are useless,' said an Indian who was in the cabin; 'that is her husband.'

These Coatas, of which I had several, are a large, black, pot-bellied monkey. They average about two and a half feet of height, have a few thin hairs on the top of their head, and look very like an old negro."

